# Impact of the 8^th^ Edition of the AJCC-TNM Staging System on Estimated Cancer-Specific Survival in Patients Aged 45–54 Years at Diagnosis with Differentiated Thyroid Carcinoma: A Single Center Report

**DOI:** 10.1155/2021/8820364

**Published:** 2021-02-22

**Authors:** Riccardo Maggiore, Francesca Perticone, Gilberto Mari, Riccardo Pasquali, Emanuele Bosi, Marina Scavini, Roberto Lanzi, Riccardo Rosati

**Affiliations:** ^1^Endocrine Unit, Department of Surgery, IRCCS San Raffaele Scientific Institute, Milan 20132, Italy; ^2^Endocrine Unit, Department of Internal Medicine, IRCCS San Raffaele Scientific Institute, Milan 20132, Italy

## Abstract

**Background:**

The 8^th^ edition of the American Joint Committee on Cancer (AJCC) staging system changed the age cutoff for risk stratification of differentiated thyroid carcinoma (DTC), downgrading patients between 45 and 54 years to stage I or II. The aim of our study was to assess cancer-specific survival (CSS) in patients aged 45–54 years, in order to document the prognostic capability of the last edition of the staging system.

**Methods:**

We retrospectively reviewed the medical records of 172 patients that from January 1^st^, 2005, to May 31^st^, 2017, were diagnosed at our institution with DTC when aged 45–54 years. We restaged patients according to the 8^th^ edition of the staging system and estimated CSS.

**Results:**

101 out of 172 patients (58.7%) were reallocated to a lower stage. Of the 101 downstaged patients, 88 (88.9%) showed a high or intermediate American Thyroid Association (ATA) risk of recurrence. We recorded no cancer-specific deaths.

**Conclusions:**

Risk of cancer-specific mortality in patients aged 45–54 years with DTC is low, supporting the prognostic capability of the 8^th^ edition of the staging system. However, we recommend to consider carefully the significant proportion of patients at intermediate or high risk of recurrence in this group of patients.

## 1. Introduction

Thyroid cancer is the most common endocrine cancer, with differentiated carcinoma (DTC) accounting for more than 90% of the overall cases. DTC usually has an indolent behavior, and although its incidence has increased in the last decades more than for any other malignancy [1], mortality rate is low (about 0.5%), with no significant changes during the last 40 years [2, 3].

DTC is the only human malignancy for which age is considered an independent prognostic factor of cancer-specific survival (CSS) by the American Joint Committee on Cancer (AJCC) staging system [4, 5]. Indeed, since 1983, an age at diagnosis of ≥45 years has been considered a predictor of mortality in patients with DTC [6], with all the patients diagnosed prior to age of 45 included in stage I or II of the disease, according to the absence or presence of distant metastases, in either case with an excellent 10-year CSS rate [7].

In the last years, however, reports by different authors confirmed a linear and gradual mortality increase in patients with DTC with age at diagnosis, although failing to identify the age cutoff able to separate patients at low and high risk of cancer-specific death [8–10], since indeed many patients older than 45 years actually show a good survival rate [11–15]. In 2016, Nixon et al. proposed a change of the age at diagnosis cutoff used in the AJCC staging system from 45 to 55 years [16], prompting a downstaging in a significant proportion of patients, without significant impact on the CSS rates of those in stages I and II [17].

The 8^th^ edition of the Tumor, Node, and Metastasis (TNM) staging system for DTC has been recently published by the AJCC [18]. One of the most significant changes compared to the previous classification is that for risk stratification, the cutoff for the age at diagnosis is increased from 45 to 55 years. Consequently, patients diagnosed in the previous years with DTC between 45 and 54 years without distant metastasis are now classified in stage I, with an expected 10-year CSS rate between 98 and 100%. According to the previous edition of the AJCC-TNM, these patients would have been included in stage I only if the tumor was <2 cm, and they had no lymph node metastasis and/or extrathyroidal extension. Otherwise, those patients would have been included in stage II, III, or IV, with a significant reduction in their expected 10-year survival [7] (Table 1).

Since its introduction in the clinical practice (January 2018), many studies have evaluated the prognostic capability of the 8^th^ AJCC-TNM edition for DTC, and some of them emphasizing that the downstaging of patients diagnosed with thyroid cancer between age 45 and 54 years may increase the number of patient in stage I with persistent or recurrent disease, therefore potentially increasing their cancer-specific mortality risk [19–22].

Aim of our study was to evaluate the impact of the 8^th^ AJCC-TNM edition on cancer-specific survival in patients diagnosed with DTC between age 45 and 54 years and treated at our institution.

## 2. Materials and Methods

We retrospectively reviewed the medical records of 172 patients with DTC diagnosed at the age 45–54 years that underwent surgery (total thyroidectomy or lobectomy) at the San Raffaele Hospital, Milan, between January 1^st^, 2005, and May 31^st^, 2017. We excluded from the analysis cases of medullary, anaplastic, or poorly differentiated thyroid carcinoma.

From the medical records, we abstracted the followings: age at DTC diagnosis, gender, tumor size, histological subtype, aggressive variants (solid, tall cells or diffuse sclerosing variant), evidence of lymph node or distant metastasis, extent of surgery (thyroidectomy or lobectomy), neck lymph node dissection (central and/or lateral), postsurgical radioiodine ablation therapy, stage according to the 7^th^ edition of the AJCC-TNM staging system, risk of tumor recurrence, locoregional or distant recurrence (if present), vital status and, in case of documented death, date and cause of death. For our entire cohort, the pathology reports were generated by the same pathologist.

Through a careful revision of surgical and pathology reports, we staged patients according to the 8^th^ TNM edition. Criteria for tumor stage assignment according to both TNM editions included age at diagnosis, tumor size, presence and degree of extrathyroidal extension, and lymph node or distant metastases.

The risk of tumor recurrence was evaluated for each patient according to the 2009 ATA risk-stratification system and divided into three cases: high (in case of distant metastases or gross extrathyroidal extension), intermediate (in the presence of lymph node metastasis, microscopic extrathyroidal extension or tumors with aggressive histology), or low (if none of the above features were present). We could not use the more recent risk stratification system proposed in the 2015 ATA guidelines because the required specific information was not reported in the pathology reports prior to 2015. Statistical analysis was performed using IBM SPSS Statistics^®^ for Windows^®^ (version 23.0). Continuous variables are reported as mean and standard deviation for normally distributed variables and as median and interquartile range for not normally distributed variables. Categorical variables are reported as proportions and frequencies.

The local ethic committee (San Raffaele Scientific Institute in Milan, Italy) approved the study protocol, and written informed consent was obtained from each patient.

## 3. Results

The characteristics of patients included in our analysis are summarized in Table 2. Mean and median follow-ups were 74 months (standard deviation 42.6) and 70 months (interquartile range 41–104), respectively.

The distribution of the patients in the different stages according to the 7^th^ and 8^th^ AJCC TNM edition is shown in Table 3. According to the 7^th^ AJCC TNM edition, 71 patients (41.3%) were in stage I, 10 patients (5.8%) in stage II, 60 patients (34.8%) in stage III, 29 patients (16.9%) in stage IVA, and 2 patients (1.2%) in stage IVC.

According to the 8^th^ AJCC TNM edition, 98.8% of the patients were in stage I and 2 patients (1.2%) in stage II. Overall, 101 out of 172 patients (58.7%) were reallocated to a stage lower than that assigned according to the criteria of the previous AJCC TNM edition.

**Figure 1:**
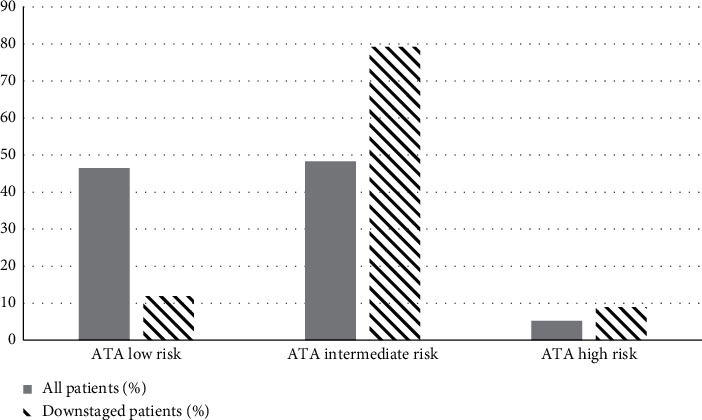
The American Thyroid Association (ATA) risk of recurrence (%) in all patients (columns on the left) and in downstaged patients according to the 8^th^ edition of AJCC-TNM staging system (columns on the right).

In our sample, we observed no cancer-related deaths. The American Thyroid Association risk of recurrence was low in 80 patients (46.5%), intermediate in 83 patients (48.3%), and high in 9 patients (5.2%). Among the 101 downstaged patients, the risk of recurrence was low in 12 patients (11.9%), intermediate in 80 patients (79.2%), and high in 9 patients (8.9%) (Figure 1).

We observed 6 cases (3.5%) of locoregional recurrence, and all of them belong to the “ATA intermediate risk of recurrence” group. All of them are downstaged according to the 8^th^ AJCC TNM edition (in 2 cases from stage III to stage I and in 4 cases from stage IVA to stage I).

## 4. Discussion

DTC is the only human malignancy with age at diagnosis included as a prognostic factor in the staging system of the American Joint Committee on Cancer (AJCC) [5]. Until the 7^th^ edition, the age of 45 years at diagnosis has been considered the cutoff discriminating between patients at lower and higher risk of mortality, with those below this limit all allocated to stage I (absence of distant metastasis) or II (presence of distant metastasis), and both stages are characterized by an excellent expected 10-year CSS [7]. More recently, however, a linear relationship between age at diagnosis and cancer mortality has been reported in a large retrospective study on patients with DTC, with no evidence of a specific cutoff able to identify differences in the survival rate [8].

In 2016, Nixon et al. suggested the age at diagnosis of 55 years as a new cutoff, resulting in the downstaging of a significant proportion of patients (about 12%) and a better separation of CSS between stages [16, 17]. Patients aged 45–54 years at diagnosis previously allocated to stage II-IV were thus shifted to stage I, with an expected 10-year CSS rate exceeding 95% [16], while those with distant metastasis were shifted from stage IVC to stage II, with an expected 10-year CSS rate of 75.5%. In 2017, Kim et al. confirmed the age at diagnosis of 55 years as a more accurate cutoff for the stratification of DTC mortality risk, allowing a downstaging of low-risk patients with no significant changes in the expected 10-year CSS rate [7]. In 2018, the 8^th^ edition of the AJCC-TNM introduced this new cutoff in the clinical practice. Since then, several studies have confirmed better accuracy of the new cutoff in predicting CSS rate in patients with DTC, while others highlighted an increased mortality risk in patients aged 45–54 years with more aggressive forms of DTC, in particular for those with locoregional or distant metastases [20, 22–27].

The main purpose of our study was to evaluate the cancer-specific survival rates in patients who were downstaged from stage IV to stage I or II by the new edition of the AJCC-TNM staging system.

Sex, age, and histological distribution of the patients included in our series is in line with the current literature [1, 2], with an incidence of aggressive variants of papillary carcinoma of 6%. According to the 8^th^ edition of the AJCC-TNM staging system, 71 patients (41.3%) of our series remained in stage I, with no significant changes of the expected CSS. On the other side, 99 patients (57.5%) were shifted to stage I from higher stages. Most of them (60 patients, 34.9%) were previously assigned to stage III, while 29 patients (16.9%) were previously assigned to stage IVA. A minority of patients, currently allocated to stage I, were previously allocated to stage II. Only two patients (1.2%), with distant metastases at diagnosis, were shifted from stage IVC to stage II.

Overall, the 18.1% of patients aged 45–54 years treated at our center for DTC presented with aggressive disease and moved from stage IV to stage I or II according to the latest edition of the AJCC-TNM staging system. The relative high proportion of patients in stage IV in our series may be explained by the fact that our institute is a high-volume tertiary center.

Despite this proportion of patients with aggressive disease, during the observation period, we observed no specific cancer-related death. This finding supports the safety of the downstaging of patients with age at diagnosis 45–54 years to stages I and II, which are characterized by a low risk of mortality.

Of note is that among patients reallocated to stages usually characterized by an indolent, not aggressive disease, the great majority (88.9%) has an intermediate or high risk of tumor recurrence, pointing out the need to implement sensitive and individualized surveillance strategies. Also in 2018 Casella et al. [28] focused on the fact that downstaged patients may have an increased risk of recurrence, dealing to the need to pay more attention on further surveillance, without modifying CSS.

At present, as already proposed by other authors [19], a promising approach to DTC follow-up could be to create further subgroups of patients, integrating the AJCC-TNM staging system with the ATA criteria for the assessment of the risk of tumor recurrence.

In conclusion, the results of our study confirm a low risk of cancer-specific mortality in patients with DTC aged 45–54 years at diagnosis, therefore supporting the prognostic capability of the 8^th^ AJCC-TNM edition. However, a tailored approach, carefully evaluating both recurrence and mortality risks, appears crucial for an efficient long-term management of these patients.

To our knowledge, this is the first study that expressly assessed the CSS rate for DTC in patients aged 45–54 years at diagnosis, confirming a low cancer-related mortality in this subgroup and supporting its inclusion in lower stages of the AJCC-TNM staging system.

Strengths of our study include the long follow-up available in a high number of patients with DTC, considering the specific subgroup of patients. Furthermore, it is a single center study in which every patient was treated with a uniform approach to surgery and every specimen received a homogeneous histology evaluation. Finally, since we are a high-volume tertiary university hospital, we had a large proportion of patients with advanced disease, making the data about mortality more reliable.

The main limit of our study is the lack of detailed information about recurrence during the follow-up. This is because many patients from the whole country access to our center for surgery and then refer to a local endocrinologist for the follow-up. Further studies addressing both recurrence and mortality could be useful to better understand the biological behavior of DTC in this subgroup of patients.

## Figures and Tables

**Table 1:** Comparison between the 7^th^ and the 8^th^ edition of the AJCC-TNM staging system for differentiated thyroid carcinoma (DTC).

	AJCC-TNM staging system, 7^th^ edition			AJCC-TNM staging system, 8^th^ edition		
	*Patients aged < 45 years at diagnosis*			*Patients aged < 55 years at diagnoses*		
Stage I	Any T	Any N	M0	Any T	Any N	M0
Stage II	Any T	Any N	M1	Any T	Any N	M1
	*Patients 45 years and older at diagnosis*			*Patients 55 years and older at diagnosis*		
Stage I	T1	N0 (Nx)	M0	T1-T2	N0 (Nx)	M0
Stage II	T2	N0 (Nx)	M0	T1-T2	N1	M0
				T3a-T3b	Any N	M0
Stage III	T3	N0 (Nx)	M0	T4a	Any N	M0
	T1-T3	N1a	M0			
Stage IVA	T4a	Any N	M0	T4b	Any N	M0
	T1-T3	N1b	M0			
Stage IVB	T4b	Any N	M0	Any T	Any N	M1
Stage IVC	Any T	Any N	M1			

**Table 2:** Demographic, clinical, and histology characteristics of the 172 patients operated for differentiated thyroid carcinoma (DTC) diagnosed at the age of 45–54 years.

Sex	
Female: 119 (69.2%)	Male: 53 (30.8%)
Histology of thyroid cancer	
Papillary: 165 (95.9%)	Follicular: 7 (4.1%)
Aggressive histology subtype	
No: 162 (94.2%)	Yes: 10 (5.8%)
Type of surgery	
Total thyroidectomy: 162 (94.2%)	Lobectomy: 10 (5.8%)
Radioiodine therapy	
Yes: 107 (62.2%)	No: 65 (37.8%)
Follow-up	
Median: 70 months	Mean: 74 months
Death from any cause	5 (2.9%)
Cancer-specific death	0

**Table 3:** Staging of the 172 patients treated for differentiated thyroid carcinoma (DTC) according to the 7^th^ and 8^th^ editions of AJCC-TNM staging system.

	No. of patients (%)	Stage (7^th^ edition)	Stage (8^th^ edition)
T1N0/Nx	71 (41.3)	I	I
T2N0/Nx	10 (5.8)	II	I
T3N0/Nx	46 (26.7)	III	I
T1-3N1aM0	14 (8.1)	III	I
T1-3N1bM0	24 (14.0)	IVA	I
T4aN0M0	3 (1.7)	IVA	I
T4aN1M0	2 (1.2)	IVA	I
T4aN1bM1	2 (1.2)	IVC	II

## Data Availability

The datasets generated and analyzed for the current study are available from the corresponding author upon reasonable request.
